# Mental health and life satisfaction among 10–11-year-olds in Wales, before and one year after onset of the COVID-19 pandemic

**DOI:** 10.1186/s12889-022-12752-6

**Published:** 2022-02-23

**Authors:** Graham Moore, Rebecca Anthony, Lianna Angel, Jemma Hawkins, Kelly Morgan, Lauren Copeland, Simon Murphy, Jordan Van Godwin, Yulia Shenderovich

**Affiliations:** 1 Centre for Development, Evaluation, Complexity and Implementation in Public Health Improvement, School of Social Sciences, Cardiff University, 1-3 Museum Place, Cardiff, CF10 3BD UK; 2 Wolfson Centre for Young People’s Mental Health, Cardiff University, Cardiff, UK

**Keywords:** Children, Mental health, Survey, COVID-19

## Abstract

**Background:**

In many countries, including in the United Kingdom (UK), COVID-19 social distancing measures placed substantial restrictions on children’s lives in 2020 and 2021, including closure of schools and limitations on play. Many children faced milestones such as transition to secondary school having missed several months of face-to-face schooling in the previous academic years.

**Methods:**

This paper examines change in mental health difficulties, life satisfaction, school connectedness, and feelings about transition to secondary school among 10–11-year-olds in Wales, UK, using data from repeat cross-sectional surveys before and after the onset of the COVID-19 pandemic. Participants were 4032 10–11-year-old schoolchildren. The first cohort completed a school-based survey in 2019 (prior to introduction of social distancing measures), and the second in 2021 (following full return to school after two rounds of school closure).

**Results:**

The percentage of children reporting elevated emotional difficulties rose from 17% in 2019 to 27% in 2021 (Odds Ratio = 1.65; 95%CI = 1.23 to 2.20). There was no evidence of increased behavioural difficulties (OR = 1.04; 95%CI = 0.73 to 1.46). There was a tendency toward declines in life satisfaction in all analyses, but this intersected the null (OR = 0.86; 95%CI = 0.70 to 1.07). Children reported a high degree of school connectedness before and after the pandemic, with no evidence of change in ratings of teacher relationships, pupil relationships or pupil involvement in school life. There was no evidence of impacts of the pandemic on children’s feelings about the transition to secondary school, with feelings becoming more positive as transition neared. Most findings were robust to a range of sensitivity analyses.

**Conclusions:**

Supporting children’s emotional recovery from the COVID-19 pandemic is a public health priority requiring urgent and effective action at multiple levels of society. Maintaining connectedness to school through the pandemic may have played a role in preventing a steeper increase in child mental health difficulties.

**Supplementary Information:**

The online version contains supplementary material available at 10.1186/s12889-022-12752-6.

## Background

In the United Kingdom (UK) before the onset of the COVID-19 pandemic, child mental health was described as having reached a point of crisis [1, 2]. In 2019, 12 % of 11–12 year old pupils in Wales, UK, reported very high scores on the Strengths and Difficulties Questionnaire [3], rising to 1 in 5 by age 15 [4]. In our 2019 primary school based survey [5], also in Wales, 8% of 10–11 year olds reported clinically significant emotional difficulties, with a similar proportion reporting clinically significant behavioural difficulties. These findings are echoed in UK-wide data focused on subjective wellbeing [6]. The Children’s Society Good Childhood Report describes a large increase in the percentage of UK children reporting unhappiness, relative to a decade earlier [7]. The authors cite increased dissatisfaction with school life and perceived appearance pressures, as potential reasons for this [7]. These trends likely also reflect structural changes; since the beginning of austerity, growth in anxiety disorders has been observed among young adults [8], and difficulties are perhaps being mirrored in children raised during this period of UK history.

It is likely that these trajectories have been amplified by COVID-19 [9]. While most children are at low risk from medical impacts of COVID-19 [10, 11], children [12] and young people [13] have borne much of the social impact of mitigations. A growing body of evidence indicates detrimental impacts of the pandemic, and control measures, on children’s health and wellbeing [14]. Educational disruption [15], coupled with worries about family members becoming unwell, disruptions to peer and family relationships, and reduced opportunities for play have likely had important impacts on children’s wellbeing and development. One systematic review found that the first wave of COVID-19 was associated with harms to children’s mental health, reductions in physical activity and growth in unmet need from health and social services [12]. The CO-SPACE study in Oxford, England, reports that child mental health difficulties increased during the first lockdown, recovering to some extent, before increasing through subsequent lockdown [16]. Internationally, pooled analyses of 29 studies since the onset of the pandemic estimates that 1 in 4 and 1 in 5 young people were experiencing elevated depressive or anxiety symptoms respectively, with higher estimates from studies conducted later in the course of the pandemic [17].

School plays vital roles in children’s development and social connectedness [18–22]. While schools cannot fully mitigate or repair harms caused by the pandemic, they have had an important role to play in supporting children, through adapting teaching practices and maintaining virtual contact with pupils throughout lockdowns and school closures [23]. Creswell and colleagues highlight that the greater increases in mental health difficulties during lockdowns in primary-school aged children relative to adolescents may in part reflect a greater tendency for younger children to feel more cut off from peers [16]. Understanding the extent to which children’s connectedness to school communities, including peers and school staff, deteriorated or continued to act as a source of wellbeing support during the pandemic, is important in understanding how children’s mental health might have been affected, and identifying areas for intervention.

As countries such as the UK begin to look beyond strong social distancing measures, understanding and addressing social harms to children is vital. In Wales, a framework for implementing a whole school approach to mental health support in schools was made available to schools in 2021, and implementation is underway [24]. In England, return to school following the second wave of COVID-19 was initially associated with policy announcements foregrounding a need to crackdown on bad behaviour [25] alongside concern over inadequately funded education recovery plans [26]. However, interventions which move away from punitive disciplinary regimes and toward fostering positive social relationships, and involving pupils in decision making, may be better for pupils’ mental health [18]. Indeed, across the UK, policy emphasis has increasingly moved toward supporting children’s emotional recovery from the pandemic [27].

Following school closures, many young people will have experienced major educational transitions, such as moving to secondary school [5], having missed several months of in-person schooling in the previous 2 school years. Life course events such as transition provide important opportunities for adaptive, and maladaptive, social development depending on how they are experienced [28]. Our primary school survey in Wales in 2019 found that prior to the pandemic, most Year 6 children reported looking forward to transitioning to secondary school, often accompanied by significant worries [5]. However, transition represents a period during which existing inequalities may widen [29], with children from poorer backgrounds or with mental health difficulties more likely to experience more negative feelings about transition to secondary school [5]. If mental health difficulties have worsened as a consequence of the pandemic, anxieties about transition to secondary school may be heightened, increasing risks of a maladaptive transition.

This paper draws on data from two surveys, conducted before the COVID-19 pandemic reached the UK (in 2019), and following full reopening of primary schools in Wales (in 2021). At the time of the second survey, schools had closed twice to most pupils for several months. We begin by examining changes in mental health outcomes, before extending to estimate changes in life satisfaction. We then explore potential social mechanisms via which the pandemic might have impacted on mental health and life satisfaction, as well as potential consequences of change in mental health. The paper addresses the following research questions:

1. Have the prevalence of emotional difficulties, behavioural difficulties and life satisfaction among 10–11-year-olds in Wales changed since the onset of COVID-19?
2. Have children’s perceptions of school connectedness changed since the beginning of the COVID-19 pandemic?
3. Have children’s feelings about the forthcoming transition to secondary school changed since the beginning of the COVID-19 pandemic?

## Methods

### Sampling and participants

This study combines two surveys, one conducted in 2019 and one in 2021. The 2019 survey aimed to replicate an earlier series of nationally representative surveys led by the research team from 2007 to 14 ([30, 31] these earlier surveys did not include measures of mental health, which were introduced in 2019). All surveys included state-maintained primary schools in Wales, which had at least one group of Year 6 (i.e. 10–11 year old) pupils. Sampling methods and response rates for the 2019 survey are described elsewhere [5]. In brief, schools who took part in our previous survey (2014) survey were approached first to take part again in 2019. Any declining school was replaced with a school in the same strata, defined by local authority and school level free school meal entitlement. The 2021 survey in turn aimed to re-engage schools who took part in our 2019 survey, and also included booster sampling in a 4 out of 22 local authorities in Wales. The latter was part of a project focused on data led action planning, including provision of school and local authority level data on pupil health and wellbeing to support action planning as an expansion of an established School Health Research Network [32] from secondary schools into primary schools. Data were weighted by local authority in all analyses to account for over-representation of some authorities. Mixed age group classes are relatively common in Wales, with Year 5 and Year 6 pupils sometimes taught in the same class; in both surveys, some schools included Year 5 pupils. However, for inclusion in the present analysis, pupils needed to be in Year 6.

### Consent and data collection

Both surveys involved 3 levels of informed consent. Schools signed an agreement to take part in a survey, and provided parents with study information and details of opt-out consent procedures, provided by the research team. Parents were provided materials 2 weeks before the survey and asked to notify the school if they did not want their child to take part. In 2019, between February and June, researchers visited each school and provided pupils with an oral description of the study within their usual classroom setting. Pupils then completed an assent form prior to participation in the survey, conducted using paper forms. Pupils were advised that participation was voluntary, that they could choose not to complete any items they did not want to and that they could stop at any time without giving a reason.

In 2021, our intention was to conduct the survey at the same time of year as in 2019; however, schools closed to most pupils from December 2020 to March 2021. We considered switching to a parent supervised home-based survey but decided this would be unlikely to yield data comparable to previous (or future) surveys. Hence, we decided to resume our survey once schools returned. Due to COVID-19 social distancing restrictions, which precluded face to face research, the 2021 survey was an online, teacher-led survey. Schools were provided with data collection protocols, tailored from our secondary school survey, which transitioned to an online teacher led survey from 2015 [33]. The survey included a frontpage asking pupils to indicate assent prior to beginning the survey. All items included an ‘I do not want to answer’ option. Five schools requested paper versions due to a shortage of IT facility to complete the online version, and these were provided and returned to the research team by courier. Ethical approval for both surveys was provided by the Cardiff University School of Social Sciences Research Ethics Committee (SREC/2700 & SREC/3874).

### Measures

#### Demographics and socioeconomic status

To measure gender, children were asked “are you a i) boy, ii) girl, iii) prefer to self-describe, iv) prefer not to say”. To measure socioeconomic status we used items from the Family Affluence Scale (FAS) developed within the WHO Health Behaviour in School-aged Children survey [34]. This includes various items on material affluence (e.g. bedroom occupancy, car and computer ownership, bathrooms in the home) which are summed to form a total score. The item on holidays in the past year was not used in 2019 due to confusion highlighted by public involvement work regarding what constituted a holiday; while asked in 2021, almost 20% did not complete this item, while among those who did, holidays were substantially less common than in previous surveys, likely due to COVID-19. Hence, a FAS score was derived from the remaining 5 items. Children were asked which adults they lived with, with response options including my mum and dad, my mum and stepdad, my dad and step-mum, my mum only, my dad only, grandparents, two mums/dads (combined with ‘mum and dad’ to form a category of ‘both parents’) with foster carers, or other adults.

### Mental health and life satisfaction

To measure pupils’ mental health symptomology, we used the ‘Me and My School Questionnaire’ [35, 36]; a 16-item measure asking children to indicate whether they ‘never’, ‘sometimes’ or ‘always’ experience a range of feelings. The scale comprises a 10-item *emotional difficulties* scale including items such as ‘I feel lonely’ and ‘I cry a lot’, ‘I am unhappy’ and a 6 item *behavioural difficulties* scale, including items such as ‘I lose my temper’ and ‘I break things on purpose’. Total scale scores are created by summing item scores, resulting in a possible range of scores of 0–20 for the emotional and 0–12 for the behavioural difficulties scales. Cut-offs indicative of potentially clinically significant difficulties have been established; a score of > = 10 is (i.e. an item average score of 1 or above) indicative of elevated symptoms on the emotional difficulties scale (10–11 borderline, > = 12 potentially clinically significant) and > =6 (i.e. an item average score of 1 or above) on the behavioural problems scale (6 borderline, > = 7 potentially clinically significant) [36]. A binary indicator is used in models in the present study (elevated vs expected levels), following descriptive analysis of the more detailed 3 category classification. The measure had good internal consistency (alpha = 0.82 for emotional difficulties in both surveys and 0.78/0.77 for behavioural difficulties in 2019/21). Our measure of life satisfaction was derived from the single item Cantril ladder [37], which asks children to select how satisfied they are with their life on a scale of 0 (worst possible life) to 10 (best possible life), with a score of 8 or above considered ‘high life satisfaction’ in the present analysis.

### School connectedness

We measured school connectedness using a series of scales used in our previous secondary school research [20]. Three questions on a 5-point Likert scale asked students to rate the extent to which they felt accepted by their teachers, that teachers cared about them as a person and that they trusted their teachers. The items demonstrated good internal consistency (alpha = 0.82/0.84 in 2019/21) and were summed to form a single measure of the perceived quality of teacher relationships. Three further questions on a 5-point Likert scale asked students about the perceived quality of peer relationships, including whether they felt students in their class enjoyed being together, were kind and helpful and accepted them as they were. The items demonstrated acceptable internal consistency and were summed to form a single measure of the perceived quality of peer relationships within school (alpha = 0.67/0.73 in 2019/21). Pupils were also asked to indicate the extent to which they agreed or disagreed with 3 statements relating to pupil involvement in school decision making, including the extent to which pupils were involved in decision making, planning activities, and that pupil ideas are treated seriously (alpha = 0.77 in 2019 and 2021).

### Feelings about transition to secondary school

Pupils were asked to select a response on a 5 point Likert scale to two questions about the forthcoming transition to secondary school, adapted from a survey by Rice and colleagues [28]. The first asked children to rate the extent to which they were looking forward to transitioning. The second asked children to indicate the extent to which they felt worried about the transition. Both were on a scale from ‘not at all’ to ‘very much’. Children were categorised as ‘looking forward to’ or ‘worrying about’ transition if they gave an answer of ‘quite a bit’ or ‘very much’ to the respective questions.

### Statistical analysis

Weighted descriptive statistics (i.e. percentages for categorical items and mean values for scale items) are presented for each variable of interest for 2019 and 2021. For the 2021 survey, to assess whether overall estimates were driven disproportionately by high estimates early in the survey period which declined as pupils settled back into school, or vice versa, we visually inspected plots of estimates over time, and used Spearman’s correlation co-efficients to estimate the association of each variables with time (i.e. days since data collection began), charting estimates by month for each variable. Regression models then compare outcomes with a binary indicator for time (i.e. 2021 = 1, 2019 = 0); these take the form of logistic regressions for most outcomes, with linear regressions for multi-item scale based variables (i.e. teacher and pupil relationships). As teacher and pupil relationships were negatively skewed, we also analysed these variables using ordinal regression as a sensitivity analysis; as findings were unchanged we report only the linear models. Survey weights were applied, and standard errors adjusted for clustering within schools, using SVY commands within Stata v15.0. Models are presented for the whole group and by socioeconomic status and gender. As a post-hoc analysis, we estimated change in mental health and life satisfaction outcomes with adjustment for school connectedness variables. As a sensitivity analysis to assess the impact of sampling differences between surveys, whole group models were re-run limited to those schools who participated in both 2019 and 2021. With the exception of the sensitivity analysis, and analyses described as post-hoc, analysis code was written prior to availability of the 2021 dataset. There was a substantially higher degree of missingness in 2021 than in 2019 for emotional (though not behavioural) difficulties, with 91 and 92% of pupils completing all items on the emotional and behavioural difficulties scales in 2019, compared to 82 and 91% in 2021. In most cases, pupils only missed one item. On average, pupils who chose not to answer one or more emotional difficulties items scored substantially higher on those completed (item mean of 0.63 vs 0.78); scores were pro-rated based on items completed, so long as at least 5 out of 10 were complete, enabling inclusion of 98% of children in analyses. For all outcomes except school connectedness (for which completion rates ranged from 84 to 89% in 2019 and from 89 to 94% in 2021), complete data were available from > 95% of participating children. Hence no further imputation was used. All models adjusted for gender, socioeconomic status and family structure.

## Results

### Response rate & sample description

Response rates for the 2019 survey are reported elsewhere [5]. In brief, 37% of schools approached took part, with 88% of pupils within participating schools completing the survey. In 2021, a total of 224 schools were approached, with 118 (53%) agreeing to take part. Following a delay to survey commencement due to school closures to most children, and the subsequent rise of the COVID Delta variant midway through the survey, only 76 schools completed the survey (34% of those approached) across 19 of Wales’s 22 local authorities, with 80% of eligible pupils within participating schools completing the survey. The sample included in this analysis includes 4032 children; 2169 Year 6 children who completed the 2019 survey and 1863 in 2021. At both timepoints, samples included an approximately equal number of children who identified as boys or girls, or neither (see Table 1). At both timepoints, approximately two-thirds of children reported living with both parents, with 1 in 10 living with a step-family and 1 in 6 with a single mother. Slightly fewer pupils reported low family affluence in 2021 relative to 2019.

**Table 1:** Raw descriptive statistics on sociodemographic factors

		Main sample (all schools)	
		2019	2021
Gender	Boy	1103 (50.9)	883 (47.4)
	Girl	1050 (48.4)	920 (49.4)
	Other	*	18 (1.0)
	Prefer not to say	*	42 (2.3)
Family structure	Both parents	1481 (68.3)	1260 (67.6)
	Stepfamily	203 (9.4)	184 (9.9)
	Single mum	346 (15.9)	289 (15.5)
	Single dad	29 (1.3)	14 (0.8)
	Grandparents	29 (1.3)	19 (1.0)
	Foster care	12 (0.6)	8 (0.4)
	Other/missing	70 (3.2)	89 (4.8)
Family affluence scale score	Low	748 (35.2)	543 (31.3)
	Medium	840 (39.5)	728 (42.0)
	High	540 (25.4)	464 (26.7)

*Suppressed as one or more category < 5

A sub-sample of 31 schools from 18 local authorities participated in both the 2019 and 2021 surveys, including 1645 pupils. This sub-sample, used for sensitivity analyses, was similar to the main sample in terms of gender and family composition, though fewer children were from high affluence families than in the main sample (see Supplementary Data Table 2).

### Weighted descriptive statistics by year

The percentage of children reporting clinically significant emotional difficulties was substantially higher in 2021 than in 2019 (13.1% vs 8.1%), with a higher percentage also reporting symptoms in the borderline range (14.3% vs 9.3%). Smaller differences were estimated for behavioural difficulties, with a slightly higher percentage in the borderline range but slightly fewer in the clinically significant range in 2021 relative to 2019 (see Table 2). The percentage of pupils reporting high life satisfaction was somewhat lower in 2021 than in 2019. Children’s scores on all school connectedness indicators were similar at both timepoints. Estimates of the percentage of pupils looking forward to transition to secondary school were marginally higher in 2021 than 2019, while the percentage reporting being worried about transition was marginally lower. Unweighted frequencies from the subsample of 31 schools who participated in both 2019 and 2021 were highly consistent with weighted estimates from the whole sample (see Supplementary Data).

**Table 2:** Frequencies and weighted percentages (or means where indicated) for all outcomes of interest

		2019	2021
Emotional difficulties	Expected	1727 (82.6)	1333 (72.6)
	Elevated	199 (9.3)	276 (14.3)
	Clinically significant	167 (8.2)	241 (13.1)
Behavioural difficulties	Expected	1818 (86.7)	1594 (85.4)
	Elevated	105 (5.1)	105 (7.5)
	Clinically significant	167 (8.2)	139 (7.1)
Life satisfaction	> = 7	1386 (68.3)	1087 (64.0)
Relationships with teachers	Mean (and 95% CI)	10.3 (10.1 to 10.4)	10.3 (10.1 to 10.4)
Peer relationships	Mean (and 95% CI)	9.1 (8.9 to 9.3)	9.1 (8.8 to 9.4)
Pupil involvement	Mean (and 95% CI)	8.7 (8.5 to 8.9)	8.7 (8.5 to 8.9)
Looking forward to transition to secondary school	Quite a bit/very much	1427 (67.5)	1315 (70.0)
Worried about transition to secondary school	Quite a bit/very much	760 (37.1)	654 (36.1)

Weighted descriptive data by family affluence and gender (Supplementary Figs. 1 and 2) indicate that across sub-groups, most outcomes changed only marginally over time. Similar *relative* increases in emotional difficulties are apparent by gender and socioeconomic status, although given the higher percentage of girls and children from lower affluence families who reported elevated emotional difficulties prior to the pandemic, somewhat larger *absolute* increases are observed for these groups. The percentage reporting elevated emotional difficulties increases from 19.5 to 33.8% for children from the lowest affluence families, compared to an increase from 11.7 to 18.5% in children from the most affluent. For girls, an increase from 20.3 to 29.5% compares to an increase from 14.4 to 21.6% for boys. While there is little evidence of an overall difference over time in behavioural difficulties, there is some signal of an increase in the percentage elevated behavioural difficulties among pupils from lower affluence families only (from 14.9 to 21.7%).

**Fig. 1:**
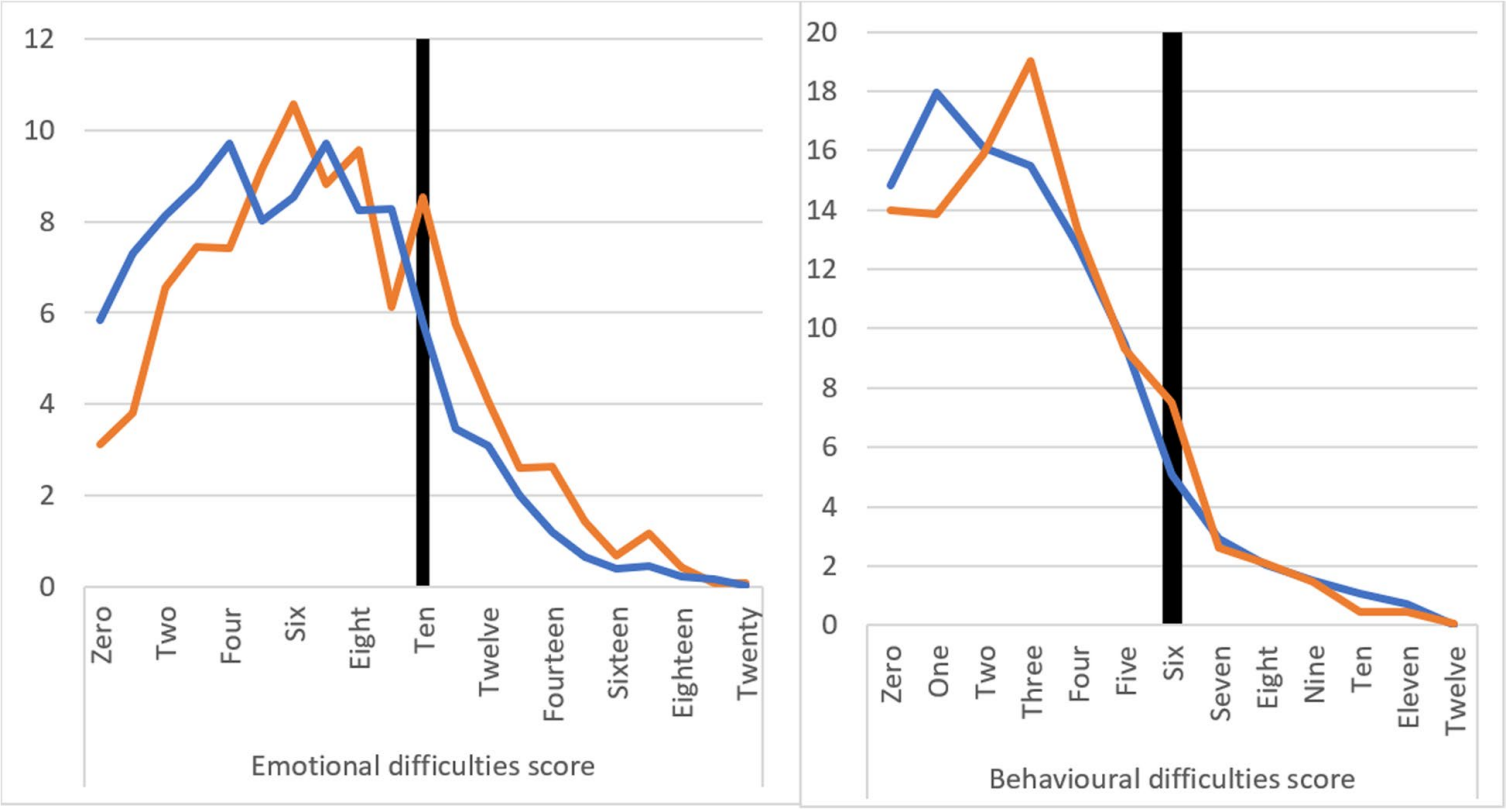
Percentage of children reporting each score for emotional difficulties (left) and behavioural difficulties (right) in 2019 (blue) and 2021 (orange). Black line indicates beginning of ‘elevated’ range

For emotional difficulties, charts of the raw scores (Fig. 1) prior to categorisation indicate a clear shift to the right of the distribution (i.e. toward higher scores) throughout the whole range, with lower scores more common in 2019 and higher scores systematically more common in 2021 (as indicated in Supplementary Fig. 3, almost every individual item on the emotional difficulties scale increased from 2019 to 2021). Scores for behavioural difficulties exhibit a less clear pattern, with some indication of movement to the right in the sub-clinical range only.

**Fig. 2:**
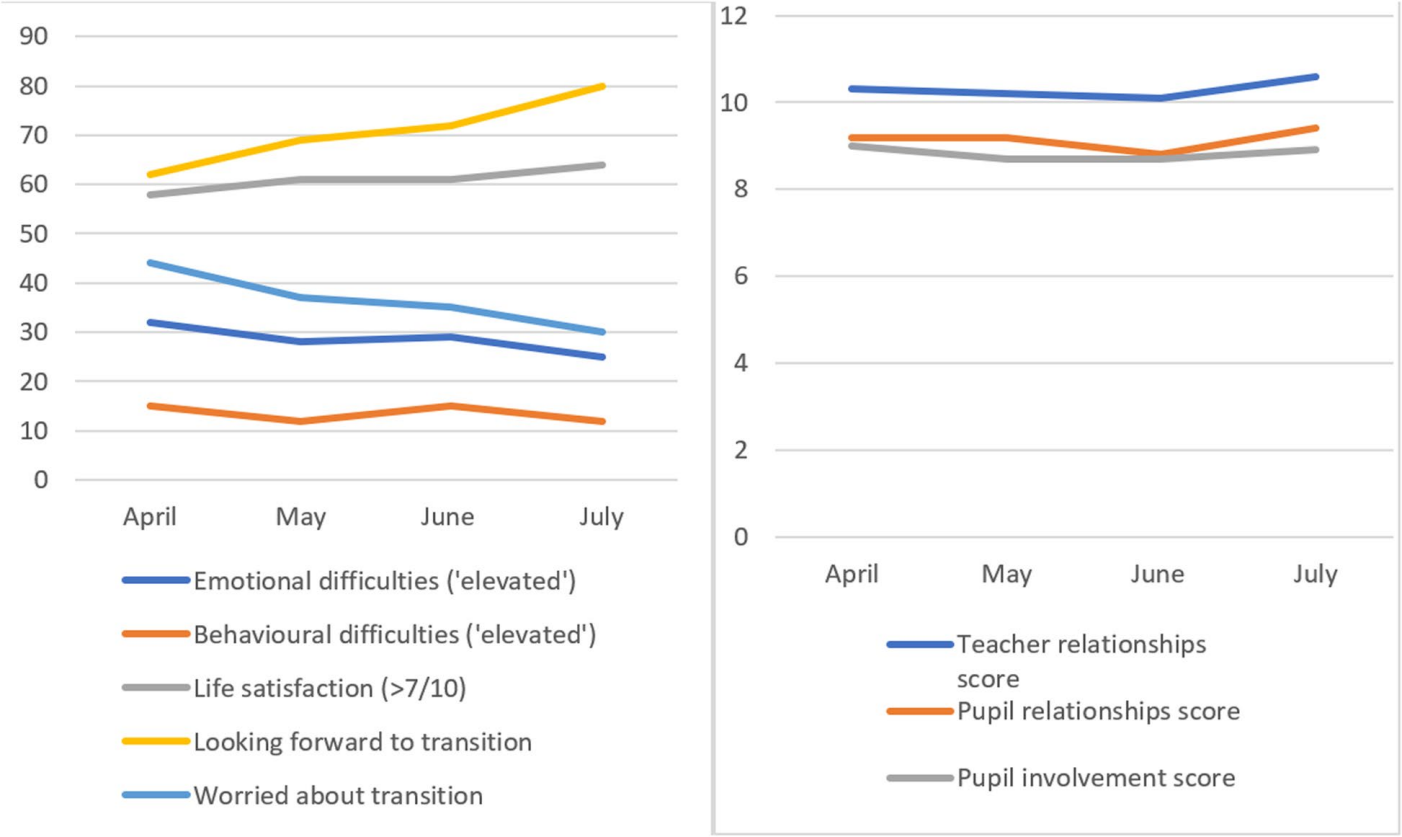
Monthly raw percentages for categorical variables (left) and mean scores for continuous variables (right) within each month of the 2021 survey

Within the 2021 survey, there was little evidence of time related change during the course of the survey for any variable of interest, with the exception of feelings about secondary school, for which there were small but significant correlations, indicative of feelings becoming more positive as the survey period progressed, and transition neared (r = 0.15; *p* < 0.001 for looking forward to secondary school; 0.07; *p* < 0.01 for worrying about secondary school). Correlations for all other variables ranged from − 0.02 to 0.02. Monthly estimates for all variables of interest are presented in Fig. 2.

### Regression models of change over time

Table 3 below indicates that the odds of reporting elevated emotional difficulties increased by approximately 65% from 2019 to 2021, with increases in all subgroups. There was little evidence of change in behavioural difficulties overall, with all estimates intersecting the null and fluctuating either side of the odds ratio of 1 across subgroups. Odds ratios were consistently in the direction of reduced life satisfaction across all models, indicating an approximately 15% reduction in the odds of reporting high life satisfaction, but all estimates intersected the null. There was no evidence of change in pupil reports of the quality of relationships with teachers, peer relationships or pupil involvement in school life. For feelings about transition to secondary school, while odds ratios were consistently in a positive direction, indicating a slight increase in the percentage looking forward to transition, all estimates intersected the null. All estimates for worries about transition also intersected the null, with odds ratios in the direction of reduced worries for all models except for lower affluence group. For behavioural difficulties, there was a graded relationships between socioeconomic status and odds ratios, with the estimate in the direction of worsening for children from poorer families, no effect for medium affluence families, and reduced difficulties for higher FAS families (consistent with descriptive data in Supplementary Fig. 2), although all intersected the null. Likewise, for peer relationships, while there was no evidence of change for poorer families, the point estimate for high FAS families was larger and in the direction of improved peer relationships during the pandemic. Again, all estimates intersected the null.

**Table 3:** Odds ratios (or coefficients) and 95% confidence intervals from logistic (and linear) regression models comparing pupil outcomes in 2021 with 2019 (2019 as the reference category)

	Whole group	Low FAS	Medium FAS	High FAS	Boy	Girl
Emotional difficulties (OR; 95% CI)	1.65 (1.23 to 2.20) *n* = 3942; p = 0.001	1.89 (1.23 to 2.90) *n* = 1241; *p* = 0.004	1.43 (1.00 to 2.04) *n* = 1541; *p* = 0.047	1.88 (1.00 to 3.54) *n* = 977; *p* = 0.051	1.65 (1.14 to 2.38) *n* = 1933; *p* = 0.008	1.61 (1.16 to 2.21) *n* = 1936; *p* = 0.004
Behavioural difficulties (OR; 95% CI)	1.04 (0.73 to 1.46) *n* = 3927; *p* = 0.84	1.37 (0.91 to 2.07) *n* = 1245; *p* = 0.14	0.95 (0.61 to 1.48) *n* = 1521; p = 0.81	0.81 (0.41 to 1.63) *n* = 979; *p* = 0.56	1.10 (0.74 to 1.62) *n* = 1926; *p* = 0.64	0.90 (0.54 to 1.50) *n* = 1919; *p* = 0.69
Life satisfaction (OR; 95% CI)	0.86 (0.70 to 1.07) *n* = 3818; *p* = 0.17	0.85 (0.62 to 1.18) *n* = 1214; *p* = 0.33	0.89 (0.67 to 1.17) *n* = 1491; p = 0.39	0.77 (0.50 to 1.19) *n* = 963; *p* = 0.24	0.86 (0.67 to 1.12) *n* = 1887; *p* = 0.26	0.87 (0.66 to 1.16) *n* = 1867; p = 0.35
Relationships with teachers^a^ (coef; 95% CI)	0.06 (−0.18 to 0.30) *n* = 3671; *p* = 0.63	0.03 (−0.33 to 0.39) *n* = 1170; p = 0.87	0.08 (−0.27 to 0.42) *n* = 1436; *p* = 0.67	0.07 (− 0.40 to 0.55) *n* = 933; *p* = 0.76	0.18 (− 0.15 to 0.52) *n* = 1810; p = 0.29	−0.04 (− 0.29 to 0.21) *n* = 1801; *p* = 0.75
Peer relationships^a^ (coef; 95% CI)	0.06 (− 0.24 to 0.37) *n* = 3541; *p* = 0.68	0.03 (− 0.33 to 0.38) *n* = 1139; *p* = 0.87	−0.09 (− 0.58 to 0.40) *n* = 1365; *p* = 0.72	0.29 (− 0.07 to 0.66) *n* = 909; *p* = 0.11	0.26 (− 0.13 to 0.64) *n* = 1774; *p* = 0.19	−0.15 (− 0.44 to 0.13) *n* = 1707; *p* = 0.29
Pupil involvement^a^ (coef; 95% CI)	0.12 (− 0.16 to 0.40) *n* = 3527; *p* = 0.40	0.23 (− 0.18 to 0.63) *n* = 1117; *p* = 0.27	−0.14 (− 0.55 to 0.27) *n* = 1374; *p* = 0.51	0.34 (− 0.07 to 0.75) *n* = 914; *p* = 0.10	0.08 (− 0.22 to 0.38) *n* = 1738; *p* = 0.59	0.12 (− 0.22 to 0.47) *n* = 1724; *p* = 0.48
Looking forward to transition (OR; 95% CI)	1.21 (0.93 to 1.57) *n* = 3937; *p* = 0.16	1.21 (0.81 to 1.79) *n* = 1256; *p* = 0.35	1.19 (0.83 to 1.72) *n* = 1533; *p* = 0.34	1.30 (0.86 to 1.96) *n* = 989; *p* = 0.21	1.23 (0.84 to 1.80) *n* = 1935; p = 0.29	1.21 (0.91 to 1.60) *n* = 1929; *p* = 0.18
Worried about transition (OR; 95% CI)	0.89 (0.72 to 1.12) *n* = 3918; *p* = 0.32	1.03 (0.80 to 1.35) *n* = 1244; *p* = 0.81	0.85 (0.63 to 1.14) *n* = 1526; *p* = 0.27	0.73 (0.41 to 1.30) *n* = 987; *p* = 0.28	0.89 (0.64 to 1.23) n = 1922; *p* = 0.47	0.89 (0.69 to 1.13) *n* = 1922; p = 0.34

^a^Linear regression

All models are adjusted for gender, family structure and SES; CI=Confidence Interval

### Sensitivity analysis

Restricting models to ‘complete cases’ for emotional difficulties (i.e. pupils who completed all 10 items) resulted in a reduced odds ratio for change from 2019 to 2021 (OR = 1.51; 95% CI = 1.13 to 2.02; *p* = 0.006) although whether pro-rated scores required 5 or 9 items to have been completed made little difference (ORs 1.59 to 1.65). For behavioural difficulties, using complete cases made little difference to estimates (OR = 1.02; 95% CI = 0.71 to 1.47; *p* = 0.91). Regression estimates without survey weights were largely consistent with the main analysis, although declines in life satisfaction became significant, while increases in the percentage of children looking forward to transition became marginally significant (Supplementary Data, Table 1). Findings for change over time from regression models limited to schools who participated in both surveys, were consistent with the main analyses (see Supplementary Data, Table 3).

### Post-hoc analysis: associations of school connectedness with mental health and life satisfaction

As a post-hoc analysis, we also adjusted regression models predicting change in emotional and behavioural difficulties and life satisfaction for school connectedness items (Table 4). Perceptions of higher quality of pupil relationships within school are associated with fewer mental health difficulties and better life satisfaction, with better teacher relationships associated with fewer emotional difficulties and better life satisfaction, with a borderline significant association with behavioural difficulties. Perceptions of pupil involvement were associated with life satisfaction though not mental health difficulties. If increases in mental health difficulties were driven by deterioration in school connectedness, we would anticipate a reduction in odds ratios for change in mental health difficulties after adjustment. Instead, in all cases, the estimate of change over time in mental health and life satisfaction increases after adjustment for school connectedness items, with change in life satisfaction becoming significant, though change in behavioural difficulties continues to intersect the null.

**Table 4:** Multivariable regression analyses of association of survey year and school connectedness items with mental health difficulties and life satisfaction (Odds ratios and 95% confidence intervals)

	Emotional difficulties (***N*** = 3124)	Behavioural difficulties (***N*** = 3122)	Life satisfaction (***N*** = 3040)
Teacher relationships (OR; 95% CI)	0.88 (0.83 to 0.94) *p* < 0.001	0.92 (0.85 to 1.01) *p* = 0.07	1.09 (1.03 to 1.16) *p* = 0.003
Pupil relationships (OR; 95% CI)	0.78 (0.70 to 0.88) *p* < 0.001	0.80 (0.71 to 0.92) *p* = 0.001	1.23 (1.14 to 1.33) *p* < 0.001
Pupil involvement (OR; 95% CI)	0.96 (0.89 to 1.05) *p* = 0.39	1.02 (0.95 to 1.09) *p* = 0.58	1.15 (1.10 to 1.20) *p* < 0.001
Time (2021 vs 2019) (OR; 95% CI)	2.01 (1.51 to 2.68) *p* < 0.001	1.22 (0.84 to 1.77) *p* = 0.28	0.72 (0.57 to 0.91) *p* = 0.007

All models are adjusted for gender, family structure and SES; CI=Confidence Interval

## Discussion

Our main findings in this study were i) substantial higher estimates of emotional, but not behavioural, difficulties in 2021 than in 2019 (prior to the pandemic) among 10–11 year olds in Wales, accompanied by a small and consistent, but non-significant, decline in life satisfaction; ii) no evidence of change in children’s connectedness to their school and iii) no evidence of change in young people’s feelings about transition to secondary school throughout the pandemic.

In our 2019 survey, approximately 1 in 6 pupils reported elevated emotional difficulties (including 8% reporting clinically significant difficulties), equating to approximately 5 pupils in an average sized UK classroom of around 30 pupils. Consistent with global estimates of the prevalence of emotional difficulties among young people [17], the percentage reporting elevated difficulties increased to more than 1 in 4 pupils by 2021 (including 1 in 8 reporting clinically significant difficulties), equating to approximately 8 pupils per classroom. This rate of increase is comparable to recent reports from England, which estimate that 17% of 6 to 16 year olds had a probable mental health disorder in 2021, compared to 12% prior to the pandemic [38]. Visual examination of distributions across the whole range for emotional difficulties indicated that pupils scores moved to a higher level throughout, with the whole symptom distribution shifting to the right. It is likely that many children who will go onto develop future mental health difficulties may be from the large number of pupils who remain in the range not considered ‘elevated’ [39].

There were comparable relative increases in emotional difficulties throughout the socioeconomic distribution and among boys and girls. However, given the higher baseline prevalence of emotional difficulties among girls and children from less affluent families, a comparable relative increase represents an amplification of pre-existing absolute inequalities between these groups. We found little evidence of increases in behavioural difficulties. While some studies find increases in behavioural difficulties during school closures [16], these fluctuate rapidly during closure and opening, and it is possible that increases in behavioural difficulties during closure had retreated by our follow up in schools. Consistent with our findings, a recent study comparing maternal reports of child mental health before and during the pandemic found increases in emotional difficulties, and in domains not covered by our measure of behavioural difficulties such as peer problems, but not in conduct problems [40]. Nevertheless, in our study, there was also some signal of increased inequality in behavioural difficulties, with increases only among children from poorer families, though this did not reach significance.

Our main findings contrast with one study, also from Wales, which reported reduced emotional and behavioural difficulties during the first lockdown [41]. It is possible that children’s mental health improved initially, but worsened as the pandemic progressed, although this explanation contrasts with data from Mental Health of Children and Young People surveys in England, which reports that most increase in children’s mental health occurred by the end of the first lockdown [38]. Notably however, the study compared a home-based survey during lockdown with previous classroom based surveys [41]. Our findings are consistent with a growing body of UK and international evidence which finds adverse population impacts of the pandemic, and social distancing measures such as school closure [40], on children’s mental health [12, 14, 16, 17].

Secondary analyses focused on potential mechanisms through which the pandemic might have impacted pupil mental health (i.e. school connectedness), and potential consequences of worsening in mental health (i.e. feelings about transition to secondary school). While in a UK wide study of children aged 10–16 years, The Children’s Society [6] report that children are more unhappy with school life than with many other domains of their life, in our study, children at the youngest end of this age range continued to report highly positive relationships with their teachers, with scores almost identical at both timepoints. We found no evidence of change in perceptions of the quality of relationships with peers in school or perceptions of involvement in school life. This finding was consistent with an earlier Welsh study which found that children’s feelings about school remained positive during the first lockdown [41]. We found that better teacher and pupil relationships were signficantly associated with better mental health and life satisfaction, consistent with earlier research [20, 42]. It is plausible that these having remained relatively stable through the pandemic may have played a role in averting steeper growth in emotional difficulties.

We also found no evidence that a cohort of children who have missed much of their final two years of in person primary school teaching were more worried about transitioning to secondary school, or looking forward to it any less, than were their counterparts prior to the pandemic. Most children were looking forward to transition, often accompanied by some worries [5]. Indeed, while we found no evidence of within survey time trends for mental health outcomes in 2021, feelings about transition to secondary school tended to become more positive as transition approached. For some, this life event perhaps provided a positive point of focus, allowing consideration of, and planning for, a future beyond the immediate difficulties of the pandemic, with feelings about transition becoming more positive as schools and pupils prepared for it through the final term.

The study benefits from large national samples of 10–11 year olds in Wales, and use of validated survey measures. Nevertheless, it has a number of important limitations. Mental health measures are limited to self report, without triangulation to reports by parents and teachers. Only two timepoints are available, and the extent to which changes represent effects of the pandemic or continuation of secular declines in child mental health cannot be established. COVID-19 social distancing measures forced changes to survey methodology, which may have influenced children’s responses in 2021. Our intention in 2021 had been to conduct a mixed mode survey in which half used a researcher led pen and paper approach and half a teacher led electronic survey. Only the latter was possible due to suspension of face to face research. Administration by teachers, while following protocols derived from our secondary surveys, might have impacted children’s responses due to children’s pre-existing relationships with teachers. Many large school-based surveys have transitioned online in recent years, with mode effect studies finding this does not bias estimates, although increases missingness [43]. While in some smaller studies, higher estimates have been observed where using online versions of child mental health measures, these have impacted behavioural difficulties as much as, or more than, emotional difficulties [35]. That large increases in our study were highly specific to emotional difficulties enhances confidence that change was not simply an artefact of survey mode. Mode effects may become smaller once a mode is no longer novel [44], and the fact the survey occurred after a year of adaptation to online learning likely mitigated these. There was higher missingness in 2021 on the emotional difficulties scale, which although perhaps a consequence of changes in method, might also reflect added sensitivity in asking about emotional difficulties during a period of trauma. School connectedness items derived from secondary school surveys exhibited moderate missingness. Further development for younger pupils may be needed and it remains possible that other facets of connectedness which may have been impacted by the pandemic were not captured. Closure of schools in early 2021 meant the survey occurred later in the school year than the 2019 survey. A large number of schools who agreed to take part in the 2021 study were unable to due to the narrowing of the data collection window due to school closures, and pressures from COVID-19. Weighting is relied upon for ensuring national representativeness of the 2021 data in particular. However, sensitivity analyses focused only on schools who participated in both surveys generated very similar estimates and led us to the same conclusions, increasing confidence that findings are not a consequence of differential response between years. We surveyed only older pupils, and it is likely that experiences of the pandemic will differ for both secondary school aged children, and younger primary school children. While some studies find larger increases in mental health difficulties in primary schoolchildren, others have reported greater mental health impacts in older adolescents [14]; these differences will be explored in our forthcoming secondary school surveys. While our sample for estimating changes in mental health difficulties among the whole population is large, some sub-group analyses likely lacked power to estimate changes.

## Conclusions

Supporting children’s emotional recovery following the COVID-19 pandemic is a major public health priority, and it will be important to draw on lessons from past global emergencies in achieving this [45]. It is likely that the mental health of many young people who experienced elevated difficulties during the pandemic will improve as and when things return to ‘normal’. However, for many, the experiences of the pandemic and associated control measures may have a lifelong impact without adequate support. While changes in emotional difficulties are observable across the socioeconomic distribution, inequalities may emerge in how fully pupils recover from experiences through the pandemic. It is unrealistic to expect schools to have fully mitigated the impacts of the pandemic on children. However, it is plausible that school connectedness having been well maintained during the pandemic played a role in preventing a steeper increase in mental health difficulties than observed here. Schools will have an important role to play in supporting children’s recovery. With behavioural difficulties having not worsened since the onset of COVID-19, while emotional difficulties have increased, an emphasis on ‘cracking down’ on perceived bad behaviour, rather than on supportive social relationships and emotional recovery, may be counter-productive. Universal interventions will be of importance in shifting the distribution of risk to children’s mental health due to COVID-19, as indicated by the substantial growth not only in clinical, but also in borderline emotional difficulties and below. However, many children may need more intensive and individualised support. Hence, investment in child mental health services, which were already under significant strain prior to the pandemic, is likely to be crucial to supporting the recovery of children experiencing significant difficulties following the pandemic. The economic impacts of the pandemic will likely have had profound effects on the mental health of many children and families, through increasing levels of poverty. Hence, continued monitoring of child mental health, and its determinants, will be critical in understanding children’s recovery as they move beyond the experience of the pandemic, the emergence of individual differences in recovery, and the effectiveness of COVID-19 recovery efforts. Supporting children’s emotional recovery will require action at multiple levels of society in the years to come.

## Supplementary Information

**Additional file 1: Figure 1.** Prevalence estimates (i.e. percentages) for each variable of interest in 2019 and 2021 by socioeconomic status (top) and gender (bottom). **Figure 2.** School connectedness scale mean scores by socioeconomic status (top) and gender (bottom) in 2019 and 2021. **Figure 3.** Weighted estimates in 2019 and 2021 for each individual item of the emotional difficulties scale of the Me and My Feelings Questionnaire (percentage saying ‘sometimes’ or ‘always’). **Table 1.** Unweighted prevalence estimates (and means) for variables of interest and estimates from regression analyses of difference between survey years. **Table 2.** Sample description for sensitivity analysis sample of schools participating in both waves (*n* = 1645 pupils within 31 schools across 18/22 local authorities in Wales). **Table 3.** Descriptive statistics and regression estimates for change in mental health, life satisfaction, school connectedness and feelings about transition to secondary school in sensitivity analysis sample (*n* = 1645 pupils within 31 schools across 18/22 local authorities in Wales).

## Data Availability

The datasets used and/or analysed during the current study are available from the corresponding author on reasonable request. Some items are restricted to eliminate risk of deductive disclosure.
