# Supplementary material for: Mental health and life satisfaction among 10–11-year-olds in Wales, before and one year after onset of the COVID-19 pandemic
Source: BMC Public Health. 2022 Feb 23;22:379. doi: 10.1186/s12889-022-12752-6 (PMC8863505; doi:10.1186/s12889-022-12752-6)
Supplement: Supplementary file 1 — Additional file 1: Figure 1. Prevalence estimates (i.e. percentages) for each variable of interest in 2019 and 2021 by socioeconomic status (top) and gender (bottom). Figure 2. School connectedness scale mean scores by socioeconomic status (top) and gender (bottom) in 2019 and 2021. Figure 3. Weighted estimates in 2019 and 2021 for each individual item of the emotional difficulties scale of the Me and My Feelings Questionnaire (percentage saying ‘sometimes’ or ‘always’). Table 1. Unweighted prevalence estimates (and means) for variables of interest and estimates from regression analyses of difference between survey years. Table 2. Sample description for sensitivity analysis sample of schools participating in both waves (n = 1645 pupils within 31 schools across 18/22 local authorities in Wales). Table 3. Descriptive statistics and regression estimates for change in mental health, life satisfaction, school connectedness and feelings about transition to secondary school in sensitivity analysis sample (n = 1645 pupils within 31 schools across 18/22 local authorities in Wales). [file 12889_2022_12752_MOESM1_ESM.docx]

**Supplementary Data**

Figure 1. Prevalence estimates (i.e. percentages) for each variable of interest in 2019 and 2021 by socioeconomic status (top) and gender (bottom)

Figure 2. School connectedness scale mean scores by socioeconomic status (top) and gender (bottom) in 2019 and 2021

Figure 3. Weighted estimates in 2019 and 2021 for each individual item of the emotional difficulties scale of the Me and My Feelings Questionnaire (percentage saying ‘sometimes’ or ‘always’)

Table 1 – unweighted prevalence estimates (and means) for variables of interest and estimates from regression analyses of difference between survey years

|  |  | **2019** | **2021** | **Odds ratio (and 95% CI) unless otherwise indicated** |
| --- | --- | --- | --- | --- |
| Emotional difficulties | Expected | 1,727 (82.5) | 1,333 (72.1) | 1.77  (1.45 to 2.16)  n=3,942; p<0.001 |
|  | Elevated | 199 (9.5) | 276 (14.9) |  |
|  | Clinically significant | 167 (8.0) | 241 (13.0) |  |
| Behavioural difficulties | Expected | 1,818 (87.0) | 1594 (86.7) | 1.02  (0.80 to 1.30)  n=3,927; p=0.88 |
|  | Elevated | 105 (5.0) | 105 (5.7) |  |
|  | Clinically significant | 167 (8.0) | 139 (7.6) |  |
| Life satisfaction | >7 | 1,386 (67.6) | 1,087 (61.4) | 0.78  (0.65 to 0.93)  n=3,818; p=0.006 |
| Relationships with teachers | Mean (and 95% CI) | 10.2  (10.1 to 10.3) | 10.3  (10.2 to 10.4) | 0.05*  (-0.17 to 0.28)  n=3,671; p=0.63 |
| Peer relationships | Mean (and 95% CI) | 9.1 (9.0 to 9.2) | 9.1 (9.0 to 9.2) | 0.06*  (-0.16 to 0.28)  n=3,541; p=0.61 |
| Pupil involvement | Mean (and 95% CI) | 8.6 (8.5 to 8.7) | 8.7 (8.6 to 8.9) | 0.13*  (-0.14 to 0.40)  n=3,527; p=0.33 |
| Looking forward to transition to secondary school | Quite a bit/very much | 1,427 (68.0) | 1,315 (71.6) | 1.21  (1.01 to 1.44)  n=3,927; p=0.04 |
| Worried about transition to secondary school | Quite a bit/very much | 760 (36.5) | 654 (35.7) | 0.94  (0.78 to 1.12)  n=3,918; p=0.48 |

*Table 2. Sample description for sensitivity analysis sample of schools participating in both waves (n=1645 pupils within 31 schools across 18/22 local authorities in Wales)*

|  |  | **2019** | **2021** |
| --- | --- | --- | --- |
| Gender | Boy | 420 (50.3) | 384 (47.4) |
|  | Girl | 410 (49.1) | 398 (49.2) |
|  | Other | * | 6 (0.7) |
|  | Missing | * | 22 (2.7) |
| Family affluence | Low | 317 (38.6) | 271 (36.4) |
|  | Medium | 312 (38.0) | 330 (44.4) |
|  | High | 192 (23.4) | 143 (19.2) |
| Family structure | Both parents | 587 (70.3) | 544 (67.2) |
|  | Step family | 77 (9.2) | 72 (8.9) |
|  | Single mum | 124 (14.9) | 130 (16.1) |
|  | Single dad | * | * |
|  | Grandparents | 12 (1.4) | 12 (1.5) |
|  | Foster carers | * | * |
|  | Other | 23 (2.8) | 45 (5.6) |

*N not reported as one or more category with <5 cases

*Table 3. Descriptive statistics and regression estimates for change in mental health, life satisfaction, school connectedness and feelings about transition to secondary school in sensitivity analysis sample (n=1645 pupils within 31 schools across 18/22 local authorities in Wales)*

|  |  | **2019** | **2021** | **OR (95% CI) unless otherwise indicated** |
| --- | --- | --- | --- | --- |
| Emotional difficulties | Expected | 659 (82.4) | 585 (72.7) | 1.68  (1.23 to 2.29)  n=1,605; p=0.001 |
|  | Elevated | 79 (9.9) | 128 (15.9) |  |
|  | Clinically significant | 62 (7.8) | 92 (11.4) |  |
| Behavioural difficulties | Expected | 694 (86.9) | 694 (86.6) | 0.97  (0.65 to 1.45)  n=1,595; p=0.87 |
|  | Elevated | 37 (4.6) | 50 (6.2) |  |
|  | Clinically significant | 68 (8.5) | 57 (7.1) |  |
| Life satisfaction | >7 | 538 (68.3) | 495 (64.7) | 0.87  (0.65 to 1.16)  n=1,549; p=0.33 |
| Relationships with teachers | Mean (and 95% CI) | 10.3  (10.1 to 10.4) | 10.2  (10.1 to 10.4) | 0.00*  (-0.32 to 0.32)  n=1,490; p=0.99 |
| Peer relationships | Mean (and 95% CI) | 9.2  (9.1 to 0.4) | 9.1  (9.0 to 9.3) | -0.10*  (-0.40 to 0.21)  n=1,440; p=0.53 |
| Pupil involvement | Mean (and 95% CI) | 8.8  (8.7 to 9.0) | 8.8  (8.6 to 8.9) | -0.07*  (-0.41 to 0.26)  n=1,437; p=0.65 |
| Looking forward to transition to secondary school | Quite a bit/very much | 553 (68.2) | 560 (70.1) | 1.17  (0.88 to 1.57)  n=1,610; p=0.28 |
| Worried about transition to secondary school | Quite a bit/very much | 277 (34.5) | 297 (37.2) | 1.06  (0.81 to 1.40)  n=1,601; p=0.65 |

*Coefficients from linear regression
